# Temperature control in sepsis

**DOI:** 10.3389/fmed.2023.1292468

**Published:** 2023-10-31

**Authors:** Marc Doman, Michael Thy, Julien Dessajan, Mariem Dlela, Hermann Do Rego, Erwann Cariou, Michael Ejzenberg, Lila Bouadma, Etienne de Montmollin, Jean-François Timsit

**Affiliations:** ^1^Medical ICU, Paris Cité University– Bichat University Hospital, Assistance Publique – Hôpitaux de Paris, Paris, France; ^2^Inserm UMR 1137 – IAME Team 5 – Decision Sciences in Infectious Diseases, Control and Care INSERM/Paris Diderot, Sorbonne Paris Cité University, Paris, France

**Keywords:** body temperature, fever, sepsis, septic shock, antipyretics, prognosis

## Abstract

Fever can be viewed as an adaptive response to infection. Temperature control in sepsis is aimed at preventing potential harms associated with high temperature (tachycardia, vasodilation, electrolyte and water loss) and therapeutic hypothermia may be aimed at slowing metabolic activities and protecting organs from inflammation. Although high fever (>39.5°C) control is usually performed in critically ill patients, available cohorts and randomized controlled trials do not support its use to improve sepsis prognosis. Finally, both spontaneous and therapeutic hypothermia are associated with poor outcomes in sepsis.

## 1. Introduction

Fever (or pyrexia) is a common feature of sepsis and septic shock. Although multiple scientific societies have defined fever as a temperature above 38.2°C (1), the actual threshold varies widely between authors (2). Fever is most often a symptom of infection but at least 25% of hyperthermia in critical care are not infectious but of tumoral, ischemic or allergic nature (3). Fever has long been considered beneficial against infection, as many germs replicate less in high-temperature environments (4). However, very high temperatures can lead to organ damage or failure.

Temperature control in sepsis, on both sides of the spectrum, has been an unresolved issue for many years (5). Recent research into fever in sepsis has isolated groups of patients with different prognoses. This, combined with a better understanding of the microbiota, may lead us to consider the correct attitude toward the treatment of fever in a more personalized way.

In this review, we will summarize recent advances on fever in sepsis from a pathophysiological and clinical point of view.

## 2. Physiopathology

### 2.1. Physiopathology and cellular changes in fever

**Figure 1:**
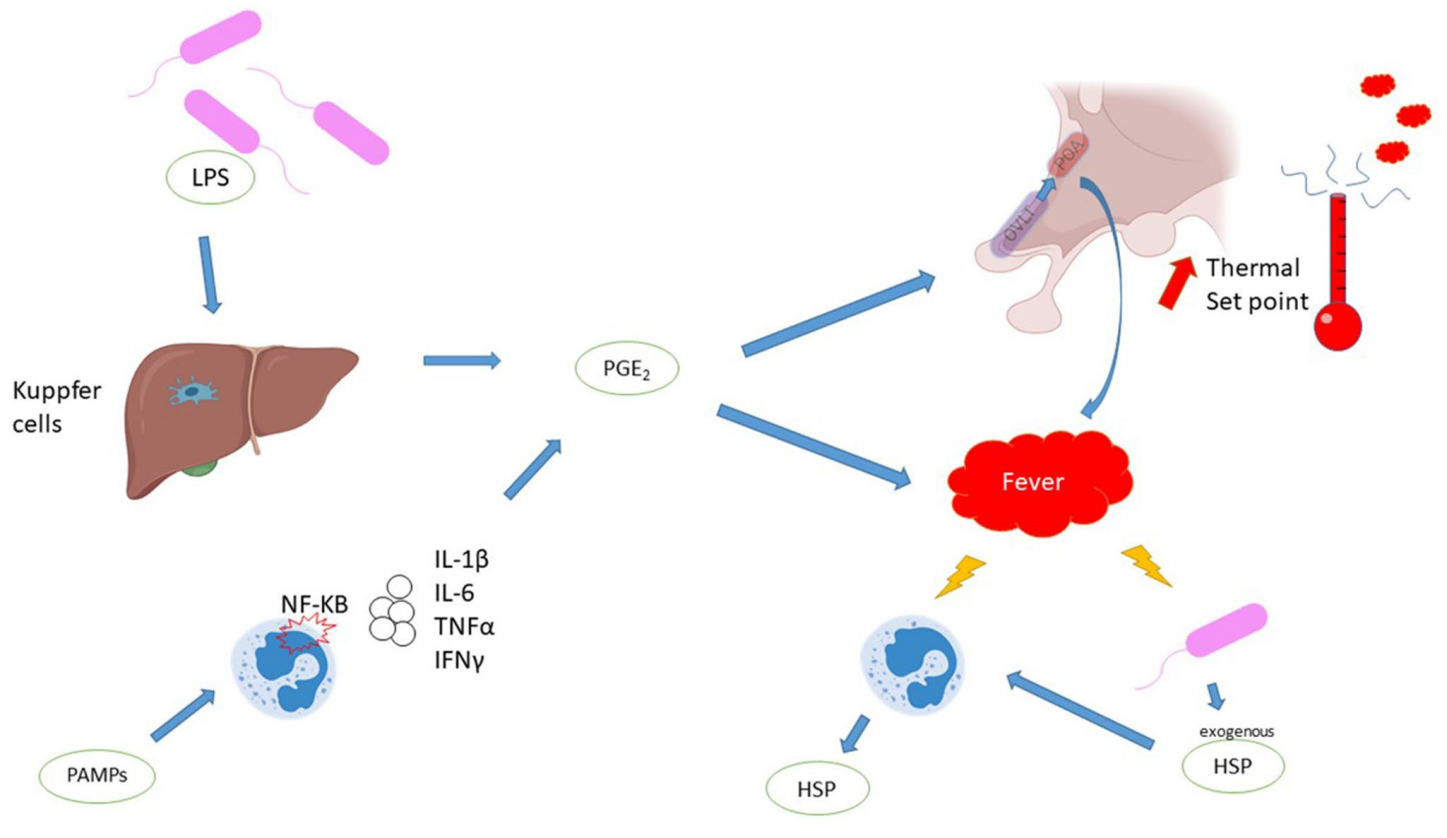
Mechanisms of fever. Comments: PGE2 is a major component of fever production. PGE2 induces a higher hypothalamus set point and promotes peripheral heat production. PGE2 is synthesized by two pathways: direct stimulation of Kuppfer cells by exotoxins, or stimulation of leukocytes via the Pathogen Associated Molecular Patterns /toll like receptor (PAMPs/TLR) pathway, leading to the production of endogenous pyrogens. Fever has multiple molecular consequences, Heat shock protein is a key response to fever is represented. Legends: HSP, heat shock protein; IFN, interferon; IL, interleukin; LPS, lipopolysaccharide; NF-KB, nuclear factor-kappa B; OVLT, organum vasculosum of the lamina terminalis; PAMPs, Pathogen Associated Molecular Patterns; PGE2, prostaglandin E2; POA, pre-optic area; TNF, tumor necrosis factor.

Body temperature is tightly regulated by the hypothalamus within a range of 0.2–0.4°C, with little circadian and ovarian variation. Homeostasis is maintained by an afferent, central and efferent system. The information comes from thermal receptors, located mainly in the skin. In the event of external or central temperature change, the hypothalamus activates efferent channels to maintain temperature by shivering, sweating or redistributing blood flow. Fever is often differentiated from hyperthermia without infection due to external heating, excessive heat production or inefficient heat loss. In hyperthermia, the hypothalamus attempts to maintain normothermia, but is overwhelmed. Fever is the result of an increase in the usual hypothalamic target or set point (usually around 37°C). The extra- and intracellular signaling leading to fever is illustrated in Figure 1, but the precise locations of action of the various molecules in the hypothalamus remain unclear (4, 6–8).

From a cellular point of view and based on *in vitro* experiments, the impact of fever is controversial. Higher temperatures inhibit the growth of *Streptococcus pneumonia* and *Haemophilus influenzae* (9, 10). In a mice pneumonia model, bacterial load was significantly lower in hyperthermic group (11). Susceptibility to antimicrobial agents is increased with temperature, especially when temperature is above 38.5°C (12). Antibiotics effects on Staphylococcal and *Pseudomonas aeruginosa* biofilms are better at higher temperatures (13, 14). On the host side, high temperatures are known to induce heat shock proteins (HSPs) which have cytoprotective properties. Fever promotes the production of HSPs by the pathogen, which in return are powerful activators of the host’s innate defense (15). Fever appears to result in an enhanced innate immune response, with greater recruitment of innate immune cells, improved neutrophil survival, better NETosis, increased production of reactive oxygen species, and reduced secretion of pro-inflammatory cytokines by neutrophils (11, 16, 17). Other data show effects on the adaptive immune system, with changes in CD4+ T cell differentiation and adaptive immune cell recruitment (18, 19).

But deleterious effects have also been described. Hyperthermia is cytotoxic, and direct cell death is described at 41°C (6). The enhanced innate immune response is accompanied by increased tissue damage, especially alveolar epithelial or neuronal damage (11, 20–22). Moreover, higher temperatures are accompanied by greater metabolism and higher oxygen consumption, a scarce resource for cells in sepsis and shock scenarios. For example, fever induces tachycardia, which increases cardiac oxygen consumption. High temperature favors arterial vasodilatation, inducing lower arterial pressure. Hyperthermia is an effective catalyst for coagulation. Underlying mechanisms could be increased tissue factor exposure from white blood cells and microparticles (23). Neutrophils, histones and NETosis are also likely to be initiators and facilitators of coagulation factor binding (23, 24).

### 2.2. Gut microbiota influence

Recently, growing knowledge in the field of microbiota has led to the question of whether gut microbiota could explain temperature heterogeneity during sepsis. A recent study isolated clusters of patients according to their temperature trajectories. Their intestinal microbiota was compared at admission to intensive care unit (ICU). The composition of the intestinal bacterial community was independently associated with individual temperature trajectories, and the mouse model reinforced the hypothesis (25). Certain taxa, such as *Lachnospiraceae* spp. may favor lower temperatures in sepsis. The same authors also demonstrated that this specific taxon is also present in lung microbiota, where it is associated with worse outcomes in critically ill patients (26).

In patients with COVID-19, fever was associated with altered intestinal microbiota, such as an increase in *Enterococcus faecalis* and *Saccharomyces cerevisiae* (27). In neutropenic fever, alteration and depletion of the gut microbiota may be involved through the loss of protective microbiota-derived metabolites (28, 29). The taxon *Akkermansia* is strongly implicated in the pathogenesis of neutropenic fever through mucus thinning (28, 30).

The interaction between the gut microbiota and the organism seems to be bidirectional. In a septic mouse model, the hosts adapted to maintain the gut microbiota. After LPS injection, small intestinal epithelial cells adapted by releasing fucose. The fucose was then consumed by the microbiota to sustain it. This phenomenon was associated with improved tolerance to infection (31).

## 3. The clinical impact and prognosis value of fever

### 3.1. Insights from non-septic situations

Heatstroke is the typical example of the harmful consequences of hyperthermia. The classic heatstroke showcases neurologic symptoms, respiratory alkalosis and in more severe cases liver dysfunction, renal failure, acute respiratory distress syndrome or even death (32). Another temperature related disease model is malignant hyperthermia. It is described as an adverse reaction during general anesthesia. The symptoms are many and have an interpatient variability (33).

In acute brain injury, fever is associated with worse outcomes (34) but target temperature management has failed show improvement in patients outcome (35, 36). Hyperthermia often occurs in cardiac arrest patients and is associated with a worse neurologic prognosis (37). Cardiac arrest management guidelines include active temperature control under 36°C in the first 24 h and fever prevention in the first 72 h (38).

Among patients admitted to the ICU without sepsis, those with the highest temperatures (>39.5°C) have the poorest prognosis (39, 40). The likely explanation is that they suffer from the negative effects of high body temperature without the alleged benefit in infectious diseases.

### 3.2. Fever in sepsis

The definition of fever in septic ICU patients varies according to ICU center worldwide with a median threshold of 38.2°C (IQR 38–38.5°C). The use of thermometers are protocolized in two third ICUs and a wide range of methods were reportedly used, with axillary, tympanic and urinary bladder sites as the most common as primary modalities (41).

Fever is reported in around 60% of septic patients in ICU (42). Approximately 10–30% of patients with sepsis may be hypothermic on admission (43–45). In a systematic review of 42 studies, mortality rate of septic patients with fever >38°C was 22.2% (CI, 19.2–25.5) which was higher, 31.2% (CI, 25.7–37.3), in normothermic patients, and it was the highest, 47.3% (CI, 38.9–55.7), in hypothermic patients (<36.0°C) (46). The-meta regression showed a strong negative linear correlation between temperature and mortality.

In contrast to non-septic patients, very high body temperature does not affect the prognosis of septic patients in the ICU (39, 40, 42, 45). The same applies to neutropenic patients admitted to the ICU, with or without hematological malignancy (47). Conversely, hypothermia is associated with a poor prognosis (40, 42, 44, 48) and a higher incidence of ICU-acquired infections (49). The deeper the hypothermia, the greater the effect. One reason for this could be a delay in the diagnosis and treatment of sepsis (50). Children, neonates and neutropenic populations also have higher mortality when hypothermic (47, 51). However, in elderly patients with sepsis, hypothermia is more frequent and not associated with mortality (52).

Interestingly, the temperature profile in sepsis can be related to outside temperature. In a German ICU population with sepsis, body temperature was independently related to outside temperature (42). High fever was more frequent when outside temperature was above 15°C, and hypothermia when it was below 4°C. This could lead to confounding bias in other studies when comparing fever and hypothermia, given that they can occur at unequal times of year. In support of this, one study shows that the incidence of nosocomial bacteremia increases with outside temperature (53).

By examining temperature trajectories, 4 phenotypes were described: rapid-resolution hyperthermia, slow-resolution hyperthermia, normothermia and hypothermia (43). Hypothermia was more frequently observed in older patients, while slow-resolving hyperthermic patients were the youngest and had the fewest comorbidities. Rapid-resolution hyperthermic patients had the lowest mortality (3%), while hypothermic patients had three times the mortality (10%). In neutropenic patients, slow-resolving hyperthermic patients have the highest mortality (54). Neutropenic patients with persistent hyperthermia could be affected by multi-resistant bacteria, fungal infections or associated with invasive devices, which could explain their poor prognosis.

Another use for temperature trajectories could be the prediction of hospital-acquired sepsis. In a case–control study, temperature changes (in terms of frequency, amplitude or progressive increase) prior to fever were an early marker of sepsis and could lead to earlier recognition of sepsis (55).

## 4. How do we control fever in sepsis? And should we?

Temperature control is variable between ICUs. A survey in the EUROBACT participants in 2012 found that temperature control management is protocolized in only 22% of the ICUs. While reported practice was to treat almost all patients with neurological impairment and most patients with acute coronary syndromes and infections, severe sepsis and septic shock, this was not the case for most patients with liver failure (41).

Target temperature management (TTM) has been a hot topic in sepsis for some time. Although there is no evidence of increased mortality in patients with very high temperatures, the authors point to the deleterious effect outside the sepsis setting as a reason to explore the effects of target temperature management in sepsis. The expected beneficial effect of TTM is a decrease in metabolism and an increase in vasomotor tone. Control of infection with antibiotics is mandatory before attempting TTM.

### 4.1. Means to TTM

#### 4.1.1. Paracetamol

The effect of paracetamol is mediated by a lowering of the hypothalamic set point, although the exact mechanism is unknown. The drug and its safety are well established. The main side effect is hepatic cytolysis and, in the most severe cases, liver failure. In terms of efficacy, the “HEAT” trial involved 700 septic patients with fever (>38°C). Patients were randomized to receive either paracetamol or placebo. The study concluded that there was no effect on ICU length of stay or mortality (relative risk 0.96, *p* = 0.84) (56). However, the study had two shortcomings: patients were febrile for a short time, and the difference in temperature between the two groups was marginal.

#### 4.1.2. NSAIDs

NSAIDs ultimately reduce PGE2 production via inhibition of cyclooxygenase. NSAIDs are widely used in ambulatory scenarios, benign and/or viral fevers (57). The most common side effect is gastrointestinal bleeding, but renal and hepatic side effects are also possible. The main obstacle in the case of sepsis is the risk of worsening the infection.

All attempts to demonstrate improved survival with NSAIDs have failed. Haupt et al. randomized 29 patients to ibuprofen or placebo in the setting of sepsis, although an antipyretic effect was demonstrated, no effect on mortality was found (58). They found no difference in secondary outcomes, including cytokine levels. Bernard et al. conducted a double blind randomized controlled trial, including 455 patients with sepsis. Although ibuprofen reduced prostacyclin and thromboxane and lowered fever, tachycardia, oxygen consumption and lactic acidosis, it did not prevent the development of shock or improve mortality (59). Morris et al. randomized 120 patients to ibuprofen or placebo. Ibuprofen lowered temperature, but had no effect on outcome (60). Finally, Memiş et al. compared 40 patients randomized to lornoxicam or placebo in severe sepsis (61). They found no difference in outcomes, biological parameters, including cytokine levels.

#### 4.1.3. External cooling

Physical methods include surface cooling and endovascular devices. Surface cooling can range from simple skin exposure or ice packs to dedicated devices (62, 63). Apart from air-circulating blankets, there is no difference in the induction of normothermia (64). Endovascular devices carry their own risks associated with central vascular access (8). Physical cooling, does not influence central control, but acts by directly eliminating heat. The hypothalamus reply will result in shivering, tachycardia and peripheral vasoconstriction, all of which are sources of discomfort for patients and may paradoxically lead to increased oxygen consumption. Shivering should therefore be avoided, and can be prevented by sedation and neuromuscular blockade (65).

Schortgen et al. conducted the main trial comparing external cooling to placebo, it involved 200 patients with septic shock. The cooling method to achieve normothermia was applied according to local procedure. The primary endpoint, the number of patients with a 50% decrease in vasopressors at 48 h, was not different (72 vs. 61%; absolute difference: 11%; 95%CI, −23 to 2) but day 14 mortality was significantly lower (19 vs. 34%, *p* = 0.013). The mortality difference was not found at day 30 (66).

### 4.2. Should we control hyperthermia?

With TTM well established in the management of cardiac arrest, intensivists gradually extended its use to septic shock. In a retrospective cohort of septic shock patients, over 75% of hyperthermic patients were treated with acetaminophen, and a small proportion even received methylprednisone (43). In addition, although it is not their intended use, renal replacement therapy and any other extracorporeal circulation therapy lower the temperature. The Surviving sepsis campaign guidelines make no mention of this topic, despite the available data.

Since hyperthermic presentation of sepsis has better prognosis, one could challenge the need to lower fever in sepsis. In an original design, preventing very high fever (T > 39°C) did not prevent organ dysfunction (67). In two studies about septic patients undergoing mechanical ventilations, better temperature control did not improve hemodynamic or respiratory conditions (68, 69). But this effect could be canceled out at very high temperatures, as shown in the retrospective study by Evans et al., in which maximum temperature > 39.4°C lost its beneficial effect on survival (70). However, in the same study, antipyretic drugs did not affect survival.

A meta-analysis of the largest trials and observational studies (1,507 patients) including drug induced and external cooling, showed that antipyretic therapy did not reduce 28-day hospital mortality in the randomized studies (relative risk, 0.93; 95% CI, 0.77–1.13; I2 = 0.0%) or observational studies (odds ratio, 0.90; 95% CI, 0.54–1.51; I2 = 76.1%) (71).

In an individual patients data meta-analysis (95% of the 1,413 included patients were infected at baseline), a more active fever management did not result in a statistically significant improvement in survival time compared with less active fever management [hazard ratio 0.91; (95% CI 0.75–1.10), *p* = 0.32] even in the subgroup with a temperature of more than 39.5°C at baseline [hazard ratio 0.76 (0.35–1.67)] (72). In a sensitivity analysis, the metanalysis was in favor of the less active temperature management in the patients mechanically ventilated with vasopressor support [*n* = 595, Hazard ratio 1.21 (1.00–1.45)].

More recently another meta-analysis including 5,140 participants, 23 trials assessing antipyretic drugs, physical cooling or both methods found similar results (65). Meta-analysis and sequential analysis of the trials showed that the hypothesis that antipyretic therapy reduces the risk of death (risk ratio 1.04, 95% confidence interval 0.90 to 1.19; I2 = 0%; *p* = 0.62; 16 trials; high certainty evidence) and the risk of serious adverse events (risk ratio 1.02, 0.89–1.17; I2 = 0%; *p* = 0.78; 16 trials; high certainty evidence) could be rejected. Similar negative results were found in studies using antipyretic agents or physical cooling.

Taking this a step further, induced hypothermia (32–34°C) in septic shock has been shown to prolong the duration of acute respiratory failure and shock and to increase 30 days mortality (73).

### 4.3. Warming hypothermia?

Since hypothermia presentation is common in sepsis, and since is it associated with higher mortality, one could advocate for actively warming patients. Active hyperthermia could lead to better organ perfusion, antibiotics concentration, and immune system activation (74–76). The available clinical data is scarce. In a Delphi method survey, a majority of physicians actively rewarmed patients (77). In another trial, normothermic septic shock patients were to receive warming of 1.5°C vs. standard of care. Induced hyperthermia was associated with better outcome (28 day survival and hospital free days) but the trial is hampered by imbalance in baseline characteristics despite randomization (78). Further studies are needed in order to evaluate warming in hypothermic and normothermic sepsis. A pilot study is in progress to assess feasibility of warming afebrile sepsis to 39°C (Clinicaltrials.gov - NCT04961151).

### 4.4. What should the bedside doctor do?

As the efficacy of TTM is not widely recognized, side effects should be avoided.

In the case of normothermic sepsis, there are no data to recommend a particular temperature range.

For hyperthermia, only the highest temperatures (T > 39.5°C) justify the use of MTT. While paracetamol is common and low-risk, external cooling carries a higher risk of chills and skin lesions. We suggest treating patients with very high temperatures (T > 39.5°C) with paracetamol and external cooling if necessary to achieve <38.2°C. Shivering should be avoided at all costs by sedation or neuromuscular blockade, and TTM should be discontinued if shivering cannot be avoided or in the presence of skin lesions.

In the event of hypothermic sepsis, rewarming should be considered and carried out gradually over several hours to avoid vasoplegia.

## 5. Conclusion

Fever is a carefully regulated mechanism, with variable consequences. In sepsis, mortality is inversely correlated with blood temperature over a wide temperature range. However, available data suggest that extreme hypo- or hyperthermia are deleterious. Despite the scientific basis for temperature control, several trials have failed to demonstrate the benefit of induced normothermia in sepsis. The control of extremely high and extremely low temperatures is usually performed in ICU to limit patients’ discomfort without definite proof.

## Author contributions

MDo: Writing – original draft, Writing – review & editing. MT: Writing – review & editing. JD: Writing – review & editing. MDl: Writing – review & editing. HR: Writing – review & editing. EC: Writing – review & editing. ME: Writing – review & editing. LB: Writing – review & editing. EM: Writing – review & editing. J-FT: Writing – original draft, Writing – review & editing.
